# Asymmetry in the Central Nervous System: A Clinical Neuroscience Perspective

**DOI:** 10.3389/fnsys.2021.733898

**Published:** 2021-12-14

**Authors:** Annakarina Mundorf, Jutta Peterburs, Sebastian Ocklenburg

**Affiliations:** ^1^Institute for Systems Medicine and Department of Human Medicine, MSH Medical School Hamburg, Hamburg, Germany; ^2^Biopsychology, Institute of Cognitive Neuroscience, Faculty of Psychology, Ruhr University Bochum, Bochum, Germany; ^3^Department of Psychology, MSH Medical School Hamburg, Hamburg, Germany

**Keywords:** brain structure, laterality, hemispheric asymmetry, psychopathology, mental health, clinical neuroscience

## Abstract

Recent large-scale neuroimaging studies suggest that most parts of the human brain show structural differences between the left and the right hemisphere. Such structural hemispheric asymmetries have been reported for both cortical and subcortical structures. Interestingly, many neurodevelopmental and psychiatric disorders have been associated with altered functional hemispheric asymmetries. However, findings concerning the relation between structural hemispheric asymmetries and disorders have largely been inconsistent, both within specific disorders as well as between disorders. In the present review, we compare structural asymmetries from a clinical neuroscience perspective across different disorders. We focus especially on recent large-scale neuroimaging studies, to concentrate on replicable effects. With the notable exception of major depressive disorder, all reviewed disorders were associated with distinct patterns of alterations in structural hemispheric asymmetries. While autism spectrum disorder was associated with altered structural hemispheric asymmetries in a broader range of brain areas, most other disorders were linked to more specific alterations in brain areas related to cognitive functions that have been associated with the symptomology of these disorders. The implications of these findings are highlighted in the context of transdiagnostic approaches to psychopathology.

## Introduction

Large parts of the human brain show structural differences between the left and the right hemisphere (Güntürkün and Ocklenburg, 2017; Esteves et al., 2020; Güntürkün et al., 2020). In general, three forms of structural hemispheric asymmetries can be distinguished: macrostructural asymmetries such as asymmetries in volume or surface of specific areas in the brain, microstructural asymmetries such as asymmetries in neurite structure, and asymmetries in gene expression (Amunts, 2010). All three forms of structural hemispheric asymmetries can be investigated in both the gray and the white matter of the brain (Hugdahl and Westerhausen, 2010; Ocklenburg et al., 2016a).

Regarding macrostructural asymmetries in gray matter, a recent large-scale study by the ENIGMA consortium analyzed asymmetries in thickness and surface area of cortical brain areas based on MRI scans of 17,141 healthy volunteers (Kong et al., 2018). The authors identified significant structural asymmetries both on the global and the local level. While the cortex was thicker in the left hemisphere, it had a larger surface in the right hemisphere. On the local level, 24 out of 34 areas (76.5%) showed significant hemispheric asymmetries for cortical thickness, with a significant leftward asymmetry more common in anterior regions. In contrast, a significant rightward asymmetry was more commonly observed in posterior regions. For surface area, 31 out of 34 brain areas (91.1%) showed significant asymmetries. Here, no clear anterior-posterior pattern was observed, but the largest leftward asymmetries were observed for language-relevant brain areas. These findings clearly show that structural hemispheric asymmetries are the rule, rather than the exception, for the structural organization of cortical brain areas. Similar findings have also been reported for subcortical brain structures. A recent large-scale multi-site study (Guadalupe et al., 2017) assessed asymmetries in the volume of seven subcortical structures (nucleus accumbens, amygdala, caudate nucleus, globus pallidus, hippocampus, putamen, and thalamus) based on 15,847 MRI scans. All seven structures showed significant structural lateralization.

These findings are not only interesting from a basic neuroscience point of view, but also in the context of clinical neuroscience (Malatesta et al., 2021; Mundorf and Ocklenburg, 2021). Indeed, several neurodevelopmental and psychiatric disorders such as dyslexia (Woodhead et al., 2021), schizophrenia (Ocklenburg et al., 2013), ASD (Floris and Howells, 2018), and ADHD (Alperin et al., 2019) have been linked to altered functional as well as structural hemispheric asymmetries (Mundorf and Ocklenburg, 2021).

Why are alterations of structural brain asymmetries and psychopathology linked? One possibility may be that variation in genes associated with mental illness is also associated with structural asymmetries in the brain. It is conceivable that genes involved in both basic brain development and specific cognitive systems linked to disorders may be relevant here. A recent study investigated the genetic architecture of structural asymmetries in surface area and thickness of cortical structures, as well as in volume of subcortical structures in the United Kingdom Biobank dataset (Sha et al., 2021). Twenty-one genetic loci were found to be significantly associated with different aspects of structural asymmetries. Functional enrichment analysis revealed that genes related to microtubules, a group of polymers involved in building the cytoskeleton, and genes expressed in the embryonic brain were particularly relevant. Importantly, the genetic variants associated with structural symmetries in the brain overlapped with genetic variants that had been associated with ASD and schizophrenia in previous studies. Moreover, stress, as one of the major transdiagnostic environmental influence factors for mental illness (Demetriou et al., 2021), has been implicated in the ontogenesis of hemispheric asymmetries (Berretz et al., 2020).

## The Relation of Structural Hemispheric Asymmetries and Neurodevelopmental and Psychiatric Disorders

As mentioned above, several neurodevelopmental and psychiatric disorders have been related to atypical functional and structural asymmetries (Mundorf and Ocklenburg, 2021). In all cases, this relationship is relative, not absolute. While patients often show more atypical asymmetries on average, there still is a considerable number of patients with typical asymmetries. One of the core questions regarding this pattern of results is why so many disorders with widely different symptoms are related to a decrease of typical hemispheric asymmetries, but almost never an increase (Mundorf and Ocklenburg, 2021). On a theoretical level, three different types of associations between structural asymmetries and psychopathology can be conceived:

### Non-specific Association

There are one or more non-specific influence factors that affect both structural brain asymmetries across the whole brain and the general risk to develop psychopathology independent of a specific diagnosis. These factors could functionally be associated with general processes of early brain and nervous system development. Alterations of asymmetries should have small effect sizes and be distributed across the whole brain without any relation to specific higher cognitive systems. For example, the results of recent large scale neurogenetic study analyzing the United Kingdom Biobank data suggested that genetic variants associated with gray matter brain asymmetry overlapped with genetic variants associated with autism and schizophrenia (Sha et al., 2021). As other studies reported direct genetic overlap between schizophrenia and autism (Morimoto et al., 2021), these findings may be indicative of a non-specific association.

### Diagnosis-Specific Association

There are no non-specific influence factors that influence both structural asymmetries and psychopathology. Instead, several specific influence factors affect both structural asymmetries and the risk for a specific disorder. These factors would also be functionally specific, i.e., related to neural networks linked to a specific disorder, e.g., to networks underlying language in language-related disorders such as dyslexia (Pinel et al., 2012).

### Symptom-Specific Association

There are influence factors related to a symptom group such as cognitive or affective issues that influence both structural asymmetries and psychopathology. This symptom group, however, is independent of a specific diagnosis. This would be in line with transdiagnostic approaches in clinical neuroscience such as the RDoC approach (Insel et al., 2010).

Differentiation between these theoretical approaches requires integration of the results of original studies on structural asymmetries in different disorders. The first approach suggests that there is a similar pattern of asymmetry reduction across different disorders that affects multiple brain areas in a diffuse way. The second approach suggests that there are individual patterns of asymmetry reduction for each disorder, with little overlap between them. Disorder-related asymmetry reductions should occur in brain areas related to the typical symptomatology of the disorders. The third approach suggests asymmetry reductions in brain areas functionally related to specific functions, but independent of a diagnosis. For example, the RDoC framework includes the domain “Cognitive Systems” (Insel et al., 2010). Different disorders have been related to cognitive deficits (Sciortino et al., 2021) and could therefore show similar reductions of hemispheric asymmetries in brain areas related to cognition such as the prefrontal cortex (Freund et al., 2021).

Taking these considerations into account, the present review article aims to give an overview of structural asymmetries in different disorders and to integrate findings across disorders to determine which of the three theoretical approaches fits the existing data the best. To this end, we review the evidence for alterations in structural asymmetries in the most widely investigated forms of mental illness.

We have searched the scientific databases PubMed and Web of Science for published studies focusing on structural asymmetries in gray and white matter in patients with neurodevelopmental and psychiatric diagnoses. The following neurodevelopmental and psychiatric disorders were included: anxiety and panic disorders, ASD, ADHD, dyslexia, MDD, OCD, PTSD, schizophrenia, stuttering, substance-related and addictive disorders. Of note, we will separately discuss studies investigating multiple disorders. To rely on robust and replicable evidence, we focus on meta-analyses and large-scale studies.

## Anxiety and Panic Disorders

Anxiety disorders are mainly characterized by persistent and excessive fear or anxiety of specific objects or situations. There are several types of anxiety disorders that differ in anxiety-inducing objects or situations, e.g., generalized anxiety disorder, panic disorder, and various phobia-related disorders (American Psychiatric Association, 2013). Anxiety disorders frequently manifest in childhood or adolescence and show a chronic-recurrent course (Kessler et al., 2010). The peak age of onset for anxiety disorders is 5.5 years (Solmi et al., 2021). The prevalence varies greatly between the different types, with an estimated prevalence of 6–12% for specific phobias and 3–5% for generalized anxiety disorder (Kessler et al., 2010). In a review analyzing the pooled prevalence for all anxiety disorders, 1-year prevalence was 10.6% and lifetime prevalence 16.6% (Somers et al., 2006).

Despite this high prevalence, anxiety disorders have not been the main focus of clinical laterality research, but there are some meta-analyses and large-scale studies that allow for at least some indirect conclusions about structural asymmetries. One meta-analysis comprising eight studies that used voxel-based morphometry investigated macrostructural gray matter changes in panic disorder (Wu et al., 2018). The authors found that patients with panic disorder showed lower gray matter volume in both left and right dorsomedial prefrontal cortex, left dorsolateral prefrontal cortex, right insula, right superior temporal gyrus, right middle temporal gyrus, and right superior orbital frontal cortex. While the asymmetries were not directly compared between patients and controls, this pattern of results suggests that hemispheric asymmetries in these areas except for the bilaterally affected dorsomedial prefrontal cortex could also be altered in panic disorder. Concerning functional implications, the authors suggested that this pattern of results reflects impairment of higher cognitive functions in panic disorder.

For social anxiety disorder, a multi-center mega-analysis of voxel-based morphometry data revealed no group differences between patients and healthy controls in whole-brain analysis, suggesting that group differences in structural asymmetries may also be unlikely (Bas-Hoogendam et al., 2017). However, a small meta-analysis of voxel-based morphometry studies in social anxiety disorder (Wang et al., 2018) including 11 studies with 470 patients and 522 healthy controls found significant group differences between patients and controls. Compared to controls, patients showed larger gray matter volumes in left precuneus, right middle occipital gyrus, and supplementary motor area, but smaller gray matter volume in the left putamen. While structural asymmetries were not assessed directly, these results imply that asymmetries in these brain areas may also be altered in social anxiety disorder.

Taken together, more studies directly assessing structural hemispheric asymmetries in anxiety disorders are needed. Indirect evidence suggests an inconsistent pattern in social anxiety disorder that is substantially different from panic disorders, suggesting diagnosis-specific alterations of structural asymmetries within the group of anxiety and panic disorders.

## Autism Spectrum Disorders

Autism spectrum disorders are a group of developmental disorders with onset in early childhood. They involve difficulties in communicating or interacting with other people, repetitive behaviors, and restricted interests (American Psychiatric Association, 2013). Children diagnosed with ASD also experience deficits in social-emotional reciprocity and non-verbal communicative behaviors. Around 1% of the general population are affected by ASD, with males diagnosed about four times more frequently than females (Werling and Geschwind, 2013).

Differences in structural asymmetries in gray matter between individuals with ASD and controls have been investigated in several different studies (Knaus et al., 2012; Joseph et al., 2014; Preslar et al., 2014; Dougherty et al., 2016; Floris et al., 2016; Richards et al., 2020). Since the results of these studies differed considerably, we will focus on the largest study on structural asymmetries conducted so far (Postema et al., 2019). In this study, conducted by the ENIGMA consortium, 54 independent datasets with 1774 individuals with ASD and 1809 controls were analyzed. The authors found a general pattern of reduced cortical thickness asymmetries in individuals with ASD compared to controls, no matter whether a specific area showed left- or right-hemispheric asymmetries. This affected medial frontal, orbitofrontal, cingulate, and inferior temporal areas. For cortical surface, alterations of structural asymmetries were observed for the orbitofrontal cortex. For subcortical structures, increased asymmetry of the volume of the putamen was observed in individuals with ASD. Effect sizes were generally small, indicating subtle rather than substantial changes in structural asymmetries in ASD. While some of the brain areas that showed altered structural asymmetries in ASD were functionally linked to typical ASD symptoms such as impairment in social-cognitive processing, others were unrelated to the symptoms associated with ASD. The authors therefore suggested that altered lateralized neurodevelopment may be a feature of ASD that affects a wide range of functionally diverse brain regions. Along these lines, alterations of structural asymmetries in ASD are likely linked to both general influence factors and specific influence factors associated with the impairment of social processes, an important symptom of ASD.

Importantly, a recent study using the data of the longitudinal EU-AIMS ASD study (European Autism Interventions - A Multicentre Study for Developing New Medications) highlighted the importance of individual symptom complexes for understanding the link between ASD and structural asymmetries (Floris et al., 2020). In this study, there was no clear group pattern of alterations of structural asymmetries in ASD. Instead, individuals with ASD showed a highly individual pattern of alterations, with some showing extreme leftward and others extreme rightward deviations. Notably, language delay as a symptom explained most of the variance in extreme rightward patterns. In contrast, core autism symptoms explained most of the variance in extreme leftward patterns. Importantly, language is lateralized to the left hemisphere in most individuals (Hirnstein et al., 2013), while comparative research points toward right-hemispheric dominance for social processing (Karenina and Giljov, 2018). Specifically, Karenina and Giljov (2018) suggested the existence of a right-hemispheric dominance for social processing through analysis of asymmetries in mother-offspring positioning in different animal species. Investigating the link between functional hemispheric asymmetries and mother-infant positioning during interactions has a long tradition in human laterality research (Bourne and Todd, 2004) and is still popular today (Packheiser et al., 2019, 2020; Malatesta et al., 2020b). Importantly, a relation between maternal cradling asymmetries and typical and atypical development has been suggested by several studies (Forrester et al., 2019, 2020; Malatesta et al., 2020a,c). Regarding ASD, an absence of cradling bias has been reported for children with ASD compared to intellectually disabled and typically developing children (Pileggi et al., 2015). Taken together, these findings imply that the structural results by Floris et al. (2020) need to be understood in the context of functional hemispheric asymmetries in symptom-relevant cognitive systems such as language and social cognition. This strongly suggests a symptom-specific relation between alterations in structural asymmetries and ASD. In line with this argumentation, Floris et al. (2020) highlighted the need for symptom-based dimensional approaches in clinical laterality research, as opposed to diagnosis-based approaches.

For structural asymmetries in white matter, one study reported generally reduced asymmetry of white matter microstructure in ASD (Carper et al., 2016), while two studies reported reduced leftward asymmetry of the arcuate fasciculus, an important tract in the language system (Wan et al., 2012; Joseph et al., 2014). The latter finding mirrors the link between language impairment in ASD and alterations of structural asymmetries in language-relevant brain areas suggested by Floris et al. (2020). So far, no meta-analysis on white matter asymmetries in ASD has been performed. A general meta-analysis of DTI studies in ASD revealed reduced microstructural integrity in the corpus callosum, the left uncinate fasciculus, and the left superior longitudinal fasciculus (Aoki et al., 2013). These findings are in line with the idea of long-distance underconnectivity in the left hemisphere. However, whether or not these findings reflect changes in white matter asymmetries needs to be investigated in future studies.

## Attention Deficit Hyperactivity Disorder

Attention deficit hyperactivity disorder is defined as having difficulties in paying attention and being impulsive and overactive (American Psychiatric Association, 2013). This neurodevelopmental disorder usually manifests in childhood but can persist into adulthood. Approximately 5% of children and 2.5% of adults are affected worldwide (American Psychiatric Association, 2013). Males are affected twice as likely as females (Ramtekkar et al., 2010). Research on hemispheric asymmetries in ADHD has been motivated by the observation that patients with right-hemispheric lesions often show attentional deficits (Heilman et al., 1991; Stefanatos and Wasserstein, 2001; Klimkeit and Bradshaw, 2006). This finding led to the idea that ADHD might be the result of a specific, right-hemispheric dysfunction (Heilman et al., 1991).

Most studies on structural gray matter asymmetries in ADHD focused on the basal ganglia and prefrontal areas since these brain areas are highly relevant for attentional processing. Several small studies reported alterations in structural asymmetries of the basal ganglia (Castellanos et al., 1994, 1996; Schrimsher et al., 2002; Uhlíkova et al., 2007; Dang et al., 2016; Paclt et al., 2016; Douglas et al., 2018), but the affected structures and the directions of asymmetry were inconsistent. Moreover, an absence of asymmetries in the thickness of the orbitofrontal cortex and inferior frontal cortex has been reported for ADHD (Shaw et al., 2009). The largest study on structural gray matter asymmetries in ADHD was recently performed by the ENIGMA consortium and included 1,933 individuals with ADHD and 1,829 healthy controls (Postema et al., 2021). Here, weak empirical evidence for alterations in basal ganglia asymmetry, but not frontal asymmetry, emerged. When all age groups (children, adolescents, and adults) were combined, no effect survived correction for multiple comparisons. When only adults were analyzed, there was a significant effect of globus pallidus asymmetry that survived FDR correction in order to account for multiple testing. Patients showed decreased leftward asymmetry. The effect size was small (Cohen’s *d* = −0.18). When children or adolescents were analyzed separately, again no effect survived correction for multiple comparisons.

Meta-analytical evidence also supports the idea that ADHD is linked to alterations of structural asymmetries in gray matter. An early meta-analysis of seven ADHD neuroimaging studies that did not specifically assess structural asymmetries reported a significant reduction of regional gray matter in the right putamen and globus pallidus in ADHD (Ellison-Wright et al., 2008). A more recent meta-analysis of eight neuroimaging datasets with an overall sample size of 324 patients with ADHD and 303 controls included lateralization analysis of gray matter volume (He et al., 2021). The authors reported that 38 (68%) of the 56 brain regions of interest showed statistically significant differences between ADHD patients and controls after FDR correction for multiple comparisons to avoid spurious findings. The areas for which significant group differences between patients and controls were observed included the putamen and globus pallidus, but also several other structures, including the inferior frontal and orbitofrontal cortex, and structures in the cerebellum and other parts of the brain. The authors concluded that ADHD is linked to widespread cortical changes in structural gray matter asymmetries.

It is not easy to come to clear conclusions from this data pattern. The often reported finding of alterations in basal ganglia asymmetry in ADHD suggests that symptom-specific factors linked to attention are relevant for the association of structural asymmetries in ADHD. However, the widespread alterations observed in the study by He et al. (2021) indicate that general factors influencing basic brain development may also play a role. It is also unclear why He et al. (2021) found so many significant associations, while there was only one significant association in the much larger dataset by Postema et al. (2021), even though both studies used FDR correction for multiple comparisons.

For structural asymmetries in white matter, results for ADHD are largely inconclusive. Studies have reported that ADHD is linked to alterations of asymmetries in the connection between the basal ganglia and frontal areas (Silk et al., 2016), in the cingulum, the inferior and superior longitudinal fasciculus, the cortico-spinal tract (Douglas et al., 2018), and in the posterior thalamic radiation (Wu et al., 2020). Moreover, ADHD has been linked to reduced rightward asymmetry in global network efficiency (Li et al., 2021). However, since none of these effects were observed in more than one study, more research on structural white matter asymmetries in ADHD is clearly needed.

## Dyslexia

Dyslexia is characterized by spelling difficulties, problems in reading word recognition, reading comprehension, and oral reading skills as well as by problems in performing tasks that require reading (Habib and Giraud, 2013). Dyslexia usually begins in early development and thus is a neurodevelopmental disorder. Prevalence is between 5 and 17% among school-aged children (Habib and Giraud, 2013). Since the main symptom of dyslexia is difficulty in reading despite normal intelligence (American Psychiatric Association, 2013), it can be assumed that left-hemispheric language networks in the brain are of direct relevance for the disorder. Therefore, it comes as no surprise that several authors have suggested an association between dyslexia and hemispheric asymmetries. The most prominent model is probably the highly influential Geschwind–Behan–Galaburda model (Geschwind and Behan, 1982; Geschwind and Galaburda, 1985a,b,c) which suggests that sex hormones affect hemispheric asymmetries and the risk to develop different disorders. However, a systematic investigation of the empirical evidence for different aspects of this highly complex model concluded that there is almost no empirical evidence supporting it (Bryden et al., 1994).

The largest structural gray matter asymmetry in the human language system has been observed in the planum temporale, a brain area located posterior to the auditory cortex that contains Wernicke’s area. In neurotypical individuals, the planum temporale shows leftward asymmetry on both macro- and microstructural levels (Ocklenburg et al., 2018). Early descriptive integration of neuroimaging studies in dyslexia reported that out of eight analyzed studies, four reported reduced asymmetry of the planum temporale due to a leftward size reduction in patients with dyslexia, while one study reported reduced asymmetry due to a rightward size increase (Shapleske et al., 1999). The authors concluded that dyslexia is associated with a decrease in leftward planum temporale asymmetry. A later study also reported that alterations in planum temporale asymmetry were related to a family history of dyslexia (Vanderauwera et al., 2018). Participants with no family risk for dyslexia were found to have more pronounced leftward asymmetry of the planum temporale than participants with a family risk for dyslexia. Apart from the study by Shapleske et al. (1999), no study as yet has performed meta-analytic integration of gray matter asymmetries in dyslexia. However, meta-analytic integration of neuroimaging studies in dyslexia without a focus on asymmetries revealed reductions of gray matter in left- or right-hemispheric brain areas that may indicate changes in hemispheric asymmetries in these areas. One study reported relative reductions of gray matter bilaterally in temporo-parietal areas and the cerebellum as well as left-sided in occipito-temporal regions of the cortex (Linkersdörfer et al., 2012). A more recent meta-analysis showed that dyslexia was associated with lower gray matter volume in the left posterior superior sulcus and the left orbitofrontal gyrus that contains pars orbitalis of Broca’s area (Eckert et al., 2016). Taken together, these studies suggest that dyslexia is likely associated with reduced leftward asymmetries of temporal and potentially also frontal language areas. These findings strongly support a diagnosis or symptom-based link between structural asymmetries and dyslexia.

This assumption is further supported by studies on white matter asymmetries in dyslexia. Meta-analytic integration of nine DTI studies revealed reduced microstructural integrity in the left arcuate fasciculus and the left corona radiata (Vandermosten et al., 2012), both of which are relevant for reading (Lebel et al., 2019; van der Auwera et al., 2021). Two subsequent studies confirmed a reduction of leftward structural asymmetry of the arcuate fasciculus (Vandermosten et al., 2013; Banfi et al., 2019), while a third study did not find a significant group difference for arcuate fasciculus asymmetry (Zhao et al., 2016).

## Major Depressive Disorder

Major depressive disorder is among the top 10 leading causes of disability in adolescents and adults worldwide (Vos et al., 2015, 2020), with a global prevalence of 6–18%. This disorder is characterized by loss of pleasure, motivation, energy, interest, appetite, and libido as well as disturbed sleep, feeling of worthlessness, reduced self-esteem, and reduced self-confidence, mostly occurring in so-called depressive episodes and present for at least 2 weeks (American Psychiatric Association, 2013). As several core processes that are disturbed in MDD show hemispheric asymmetries, such as self-perception and emotional processing, functional hemispheric asymmetries have been widely investigated in MDD patients (de Aguiar Neto and Rosa, 2019).

Compared to the large number of published studies on functional hemispheric asymmetries, only a few articles on structural hemispheric asymmetries in MDD have been published. Earlier small-scale studies reported inconsistent results on alteration of structural gray matter asymmetries in mood disorders (Kumar et al., 2000; Liu et al., 2016). In contrast, the largest published study on this topic reported no differences between MDD patients and controls regarding thickness and surface asymmetries in cortical gray matter and subcortical structures (de Kovel et al., 2019). For white matter asymmetries, we were unable to identify any published studies that investigated MDD patients.

## Obsessive–Compulsive Disorder

Obsessive–compulsive disorder is marked by the presence of obsessions (recurrent thoughts, urges, or images) and/or compulsions (repetitive behaviors or mental acts) in an excessive or repeated manner, occurring beyond the developmentally appropriate period of the affected person (American Psychiatric Association, 2013). The affected person frequently suppresses the occurring obsessions by performing certain compulsions (American Psychiatric Association, 2013). The mean age of onset for OCD is 14.5 years (Solmi et al., 2021), but 25% of males affected by OCD show an onset before the age of 10 years (American Psychiatric Association, 2013). The disorder has a 12-month prevalence of 1.1–1.8% (American Psychiatric Association, 2013).

Obsessive–compulsive disorder has not been a traditional focus of clinical laterality research, but recently a large-scale study by the OCD Working Group within the ENIGMA consortium investigated structural gray matter asymmetries in OCD in 16 pediatric and 30 adult datasets (Kong et al., 2020). The authors assessed asymmetries in thickness and surface of different cortical areas, as well as asymmetries in the volume of different subcortical structures. In the analysis of the 16 pediatric datasets, significant differences between OCD patients and controls reflected increased leftward asymmetry of the thalamus and reduced leftward asymmetry of the pallidum. No differences between patients and controls were observed in the analysis of datasets containing data from adult participants. This suggests that there is no strong relation between OCD diagnosis and altered structural asymmetries.

For white matter asymmetries in OCD, a recent meta-analysis of 17 TBSS datasets revealed that OCD patients showed reductions of white matter integrity in the left orbitofrontal cortex as well as in the genu of the corpus callosum (Hu et al., 2020). Interestingly, fractional anisotropy of left orbitofrontal white matter showed negative correlations with symptom severity and illness duration. As the orbitofrontal cortex has been linked to the symptoms that OCD patients experience (Bokor and Anderson, 2014), this finding is in line with a symptom-specific link between alterations in white matter structural asymmetries and OCD.

## Posttraumatic Stress Disorder

Posttraumatic stress disorder can occur in individuals who experienced traumatic events (Bisson et al., 2021). Typical traumatic events are war, natural disasters, mass catastrophes, and sexual abuse (Andreasen, 2010). PTSD is characterized by an ongoing fear-related reaction whenever the individual is re-experiencing or remembering the traumatic event (American Psychiatric Association, 2013). PTSD patients typically avoid situations, people, or memories associated with the event, and sometimes unwillingly re-experience the event. Moreover, alterations in cognition and mood concerning the event or oneself, as well as changes in arousal and reactivity, e.g., angry outbursts or feeling tense, are typical symptoms (American Psychiatric Association, 2013). PTSD has a global lifetime prevalence of approximately 8% and, depending on the country, a 12-month prevalence varying between 1 and 9% (Atwoli et al., 2015). Since stress has been suggested as a factor that may influence both the risk to develop PTSD and the ontogenesis of hemispheric asymmetries (Berretz et al., 2020), hemispheric asymmetries in brain structure have been investigated in several different studies (Ocklenburg et al., 2016b).

On meta-analytical level, earlier studies showed somewhat contradictory results. While a meta-analysis of 13 studies focused on the hippocampus reported bilateral, rather than asymmetric, volume reductions in the hippocampus in PTSD (Smith, 2005), a more recent whole-brain meta-analysis found volume reduction in PTSD specifically in the left hippocampus and the left temporal lobe (Kühn and Gallinat, 2013). Greater volume reduction of the left compared to the right hippocampus was later confirmed by two larger PTSD meta-analyses (Li et al., 2014; O’Doherty et al., 2015), among other study-specific effects. Functionally, the hippocampus is a key structure in a larger fear learning and memory network that is highly relevant for the typical symptoms experienced by PTSD patients (Harnett et al., 2020). This suggests that a symptom- or diagnosis-based approach is optimally suited to understand the relation of PTSD and alterations in structural gray matter asymmetries.

For white matter, a recent large-scale study by the ENIGMA consortium reported that PTSD patients compared to controls showed significantly lower microstructural integrity of the segment of the corpus callosum that connects the two hippocampi (Dennis et al., 2019). However, no alterations of asymmetries in larger intrahemispheric white matter pathways were described.

## Schizophrenia

Schizophrenia is one of the most widely investigated psychiatric disorders in the context of hemispheric asymmetries. With 0.3–0.7% of the global population affected at some point in their lives (van Os and Kapur, 2009), schizophrenia is characterized by two main symptom categories: positive and negative symptoms. Delusion of thought, hallucinations, speech disorders, and motor problems are positive symptoms. In contrast, anxiety, social withdrawal, flat affect, odd behavior, apathy, and self-neglect are defined as negative symptoms (Elert, 2014). Investigating hemispheric asymmetries has a long history in schizophrenia research, the main idea being that left-hemispheric dysfunction could lead to schizophrenia (Lohr and Caligiuri, 1997). One early theory linking hemispheric asymmetries and schizophrenia specifically suggested that temporal lobe epilepsy of “the dominant” hemisphere could lead to psychosis (Flor-Henry, 1969).

Regarding cortical gray matter asymmetries, two early meta-analyses found a significant decrease of the typical leftward asymmetry of the planum temporal in schizophrenia (Shapleske et al., 1999; Sommer et al., 2001). The meta-analysis by Sommer et al. (2001) additionally reported reduced asymmetry of the Sylvian fissure. Importantly, it was shown that individual decrease of lateralization in the planum temporale correlated positively with symptom severity (Oertel et al., 2010), suggesting a direct link between atypical lateralization in this brain structure and schizophrenia symptoms.

This indicates that symptom-specific factors, for example, related to the common schizophrenia symptom of auditory verbal hallucinations (Hugdahl and Sommer, 2018; Bless et al., 2020), link structural gray matter asymmetries and schizophrenia. Interestingly, a recent large-scale study on genetic effects on planum temporale asymmetry in participants from the United Kingdom Biobank (which were from the general population) found no significant genetic correlations of asymmetry in the planum temporale with schizophrenia, ASD, or ADHS (Carrion-Castillo et al., 2020). This suggests that genetic factors do not play a large role in the relationship of structural asymmetries and schizophrenia, but that other influence factors such as disorder-specific brain plasticity processes may link the two traits (Bishop, 2013). Interestingly, altered planum temporale asymmetry has also been implicated in dyslexia (see above), suggesting an overlap in alterations of structural asymmetries between these two very divergent disorders. On the subcortical level, a recent large-scale study on subcortical structural asymmetries in schizophrenia found leftward asymmetry of the globus pallidus in patients with schizophrenia that was absent in controls (Okada et al., 2016). The authors concluded that this alteration of asymmetry may be functionally linked to neural networks associated with executive functions (which are commonly impaired in schizophrenia), including the basal ganglia and frontal areas.

Regarding white matter asymmetries, two studies found alterations of asymmetries in the uncinate fasciculus (Kubicki et al., 2002; Miyata et al., 2012). Moreover, a meta-analysis of white matter neuroimaging studies in schizophrenia comparing schizophrenia patients who experienced auditory verbal hallucinations with healthy controls reported reduced fractional anisotropy in the left arcuate fasciculus in schizophrenia patients, albeit without specifically assessing asymmetries (Geoffroy et al., 2014). Another study focused on white matter asymmetries found reduced leftward asymmetry of the arcuate fasciculus in schizophrenia (Abdul-Rahman et al., 2012). Importantly, a very recent study showed that schizophrenia patients who experience auditory verbal hallucinations show differences in structural white matter asymmetries compared to patients who do not experience auditory verbal hallucinations (Beresniewicz et al., 2021). The authors found significantly reduced leftward white matter asymmetries in patients experiencing auditory verbal hallucinations compared to patients who do not experience auditory verbal hallucinations in two clusters, both located in the superior longitudinal fasciculus. The superior longitudinal fasciculus is a larger pathway connecting frontal and temporal cortices and contains the arcuate fasciculus (Gonzalez et al., 2021). Moreover, another recent study showed that schizophrenia patients experiencing auditory verbal hallucinations had longer leftward arcuate fasciculus fiber tracks than schizophrenia patients who do not experience auditory verbal hallucinations (Falkenberg et al., 2020).

It is reasonable to assume that these findings are related to a dysfunction of the language network that is especially prominent in schizophrenia patients who experience auditory verbal hallucinations. Auditory verbal hallucinations are a major symptom of schizophrenia, and it has been estimated that they occur in about 70% of schizophrenia patients (McCarthy-Jones, 2012; Fuentes-Claramonte et al., 2021). Auditory verbal hallucinations have been suggested to reflect aberrant lateralized speech perceptions (Hugdahl et al., 2012). These findings thus strongly suggest that a symptom-based approach does reflect the relationship between schizophrenia and alterations of structural white matter asymmetries better than a non-specific or a diagnosis-based approach, as has been suggested for functional language lateralization in schizophrenia (Ocklenburg et al., 2015).

## Stuttering

Stuttering is defined by disrupted fluency and time patterning of speech that does not match the individuals’ age (American Psychiatric Association, 2013). As a neurodevelopmental disorder, symptom onset usually begins during early development between 2 and 7 years of age (American Psychiatric Association, 2013). Even though 70–80% of affected children recover during development, stuttering can persist throughout life with approximately 1% of children and adolescents, and 1% of adults (0.8% men, 0.2% women) affected worldwide (Neumann et al., 2017).

As stuttering is marked by disrupted speech processes, hemispheric asymmetries in language-related brain areas have frequently been the focus of studies in patients suffering from stuttering. Results were, however, largely inconsistent. For the planum temporale, two studies reported reduced leftward gray matter asymmetry in stuttering (Foundas et al., 2001, 2004), while two more recent studies did not find this association (Cykowski et al., 2008; Gough et al., 2018). These findings indicate that the link between stuttering and planum temporale asymmetry is weak at best. Other studies in small cohorts reported reduced prefrontal and occipital asymmetries in stuttering (Foundas et al., 2003), as well as alteration of asymmetries in the caudate nucleus (Foundas et al., 2013) and brain torque (Mock et al., 2012).

For white matter asymmetries, only one small-scale study in 10 adults who stuttered and 10 controls has been published (Jäncke et al., 2004). In this study, individuals who stuttered did not show a leftward white matter asymmetry in the auditory cortex that was observed in controls. Moreover, stuttering was associated with white matter volume increases in several right-hemispheric structures related to language, but also further prefrontal and sensorimotor areas. While these findings provide preliminary evidence for changes in white matter asymmetries in stuttering, replication in larger cohorts is needed due to the small sample size in this study. Taken together, new large-scale studies and, if possible, integration of existing data, e.g., in the form of voxel-based ALE meta-analysis, is needed before any final conclusions on alterations of structural asymmetries in stuttering can be drawn.

## Substance-Related and Addictive Disorders

Substance-related and addictive disorders are divided into two main classes: substance-related addiction and non-substance-related addiction, with substantial activation of the reward system as a common factor leading to neglect of everyday activities (American Psychiatric Association, 2013). Addictive disorders are marked by compulsive use of a substance or need for a behavior irrespective of harmful consequences. Further symptoms of these disorders are craving, inability to reduce or quit, and continued use regardless of induced problems, tolerance, investing a great deal of time and effort to obtain, use, or recover from the substance or behavior (American Psychiatric Association, 2013). Global estimated prevalence is around 18.4% for heavy alcohol use, 15.2% for daily tobacco smoking in adults, and less than 4% for illicit drugs as reviewed by Peacock et al. (2018). With these numbers, substance-related and addictive disorders are amongst the most prevalent mental disorders.

Earlier studies on structural gray matter asymmetries in substance-related and addictive disorders had small sample sizes and diverging results (Jung et al., 2007; Medina et al., 2007; Zhu et al., 2018). We therefore focus on the largest study on this topic, a recent mega-analysis of 22 structural MRI datasets performed by the ENIGMA Addiction Working Group (Cao et al., 2021). In this study, the authors compared cortical and subcortical asymmetries between 1796 patients addicted to either alcohol, nicotine, cocaine, methamphetamine, or cannabis and 996 controls not suffering from an addictive disorder. They found that, on average, patients showed reduced rightward volume asymmetry of the nucleus accumbens, and concluded that disrupted structural asymmetry in this brain area might be a characteristic of addiction. The nucleus accumbens is a central component of the reward circuit in the brain (Teague and Nestler, 2021). Since altered reward processing is a central component of substance-related and addictive disorders, this finding suggests that diagnosis-specific risk factors or symptom-specific risk factors, but not non-specific risk factors, link gray matter asymmetries and this group of disorders.

Structural asymmetries in white matter have not been a major focus in research on substance-related and addictive disorders. A recent meta-analysis on white matter structure in cocaine use disorder reported lower fractional anisotropy in the genu of the corpus callosum in cocaine users compared to controls, but not specific left- or right-hemispheric alterations (Suchting et al., 2021). Additionally, a DTI study in patients addicted to alcohol (Schulte et al., 2010) reported degeneration of posterior callosal fibers and left posterior cingulate fibers in patients compared to controls. In general, more, and larger studies on white matter asymmetries in substance-related and addictive disorders need to be performed before any final conclusions can be drawn. It is also important to specifically conduct more longitudinal studies. In studies with one-time cross-sectional data collection, it is not possible to assess whether altered structural asymmetries represent a potential risk factor to develop a disorder or are themselves due to impairments provoked by persisted substance abuse in itself, like asymmetric brain atrophy.

## Studies Investigating Multiple Disorders

In addition to the previously described studies focusing on structural asymmetries in one disorder, one study has also investigated structural brain asymmetries across different groups of disorders. The study, an ALE meta-analysis, was focused on schizophrenia, bipolar disorder, and MDD (Huang et al., 2021). The authors analyzed gray matter asymmetries based on data from 169 clinical neuroimaging studies. Overall, 3517 schizophrenia patients, 1575 bipolar disorder patients, 3280 MDD patients, and 9733 controls were included, making this a well-powered study.

When analyzing regional differences between patients and controls independent of asymmetries, the authors found that the right parahippocampal gyrus and the left superior frontal gyrus showed similar alterations across all three disorders. Based on the functions of these brain areas, the authors concluded that working memory impairments in the three disorders may be influenced by similar structural alterations. However, when specifically assessing differences in structural asymmetries between patients and controls, the authors did not identify any common patterns. While MDD was linked to asymmetrical alterations with a rightward bias, schizophrenia and bipolar disorder were linked to asymmetrical alterations with a leftward bias, but in different brain areas.

These findings suggest that while there are common neurobiological alterations in MDD, schizophrenia, and bipolar disorders, alterations in structural asymmetries are not among them. Therefore, the results of this study do not support the idea that there are non-diagnosis-specific factors that are associated with both structural asymmetries and the risk to develop mental illness. Instead, they support the idea that diagnosis- or symptom-specific factors link structural asymmetries and mental illness.

## Discussion

The present review aimed to determine whether psychopathology and structural asymmetries in the brain are more likely linked by non-specific factors that generally affect the risk to develop a mental disorder or factors that affect the risk to develop a specific disorder or a specific symptom. While the results for ASD at least partly suggest a potential influence of non-specific factors with widespread impact on many different brain structures, the results for most other disorders suggested that symptom- or diagnosis-based factors linked to cognitive systems associated with disorder-specific symptoms may be better suited to explain the relation of psychopathology and structural asymmetries. For MDD, notably, no differences in gray matter asymmetries were observed. While some overlap in affected brain regions was observed between disorders, most notably for dyslexia and schizophrenia, most altered asymmetries were highly disorder-specific and typically related to the symptoms related to each disorder. For most disorders, the empirical evidence does not currently allow us to disentangle whether symptom-based or diagnosis-based approaches are better suited to explain the association of structural asymmetries and psychopathology. However, the results for schizophrenia patients with and without auditory verbal hallucinations make a strong argument for a symptom-based association. This suggests that empirical studies investigating differences in structural asymmetries between patients with the same diagnosis, but different symptoms are highly important to further advance clinical laterality research.

A recent opinion article argued that understanding altered laterality in different psychiatric and neurodevelopmental disorders is one of the core challenges for laterality research in the 2020s (Ocklenburg et al., 2021). The current review identified several critical gaps in the existing research literature on structural asymmetries and psychopathology that need to be filled to overcome this challenge.

One major gap that became evident is the fact that almost all studies linking structural asymmetries and psychopathology focused only on macrostructural asymmetries. Differences between patients and controls in microstructural asymmetries and gene expression asymmetries remain largely unexplored. While investigating the link between psychopathology and gene expression asymmetries will rely largely on animal models (Mundorf et al., 2020a, 2021a), neuroimaging techniques to investigate microstructural asymmetries *in vivo* in humans, such as NODDI (Zhang et al., 2012), are readily available. Since the first studies have already used NODDI to investigate microstructural asymmetries in healthy participants (Schmitz et al., 2019; Mundorf et al., 2021b) and linked them to cognitive phenotypes (Ocklenburg et al., 2018), application in clinical cohorts will be a logical next step.

Another major gap is the lack of cross-disorder studies investigating structural asymmetries. While the study by Huang et al. (2021) was an important step, more large-scale studies comparing structural asymmetries in a wider range of mental disorders are needed. Along these lines, more transdiagnostic studies, focusing not on diagnoses but investigating the relation of structural asymmetries and psychopathology in transdiagnostic frameworks such as RDoC (Insel et al., 2010), are warranted. While an RDoC perspective has been used in a study on functional asymmetries (Nusslock et al., 2015), it has not been used in research on structural asymmetries so far. Doing so would allow researchers to disentangle to a greater extent whether diagnosis- or symptom-specific factors link structural brain asymmetries and psychopathology.

Similar to the described lack of cross-disorder studies, there is also a lack of studies that investigate structural asymmetries in gray and white matter while taking into account all relevant functional asymmetries. For example, it has been suggested that the association between handedness and brain structural asymmetries needs further investigation (Guadalupe et al., 2014; Budisavljevic et al., 2021). Moreover, recent research highlights the importance of understanding individual hemispheric segregation, i.e., the individual pattern of distribution of different lateralized functional networks in the brain (Vingerhoets, 2019; Gerrits et al., 2020). Functional asymmetries may be shaped by structural asymmetries, but also affect structural asymmetries *via* processes like use-dependent plasticity. Therefore, integrating individual patterns of functional hemispheric integration into research designs may be highly relevant to further understand the link between structural asymmetries and neurodevelopmental and psychiatric disorders.

Last, but not least, more studies on structural asymmetries in animal models of mental disorders are needed to gain a more mechanistic understanding of why asymmetries in brain structure and psychopathology are linked. Using a preclinical animal model for early life stress, we recently analyzed the consequences of stress exposure on turning asymmetry in rats and found that prolonged early life stress leads to atypical leftward asymmetry in the elevated plus-maze test (Mundorf et al., 2020a). When investigating turning behavior in a maternal immune activation animal model for schizophrenia in rats, we furthermore found not only atypical turning behavior in the experimental condition but also an effect of age on turning asymmetry, with adolescents showing a more pronounced asymmetry than adults after maternal immune activation (Mundorf et al., 2021a). Similar experiments could also be conducted using established clinical models of other mental disorders (Mundorf et al., 2019, 2020b; Bölükbas et al., 2020; Lopez and Bagot, 2021) and with a focus on structural asymmetries.

## Conclusion

In the present review article, we gave an overview of research on structural asymmetries in the brain in different mental disorders. Focusing on large-scale studies and meta-analyses, we revealed that alterations in structural hemispheric asymmetries are widespread across almost all investigated disorders. Cross-disorder overlap in these alterations was minimal. This suggests that diagnosis- or symptom-specific factors link asymmetries in brain structure and psychopathology, and not unspecific factors that generally increase the risk to develop mental illness. Further research is needed to determine to what extent diagnosis- or symptom-specific factors link asymmetries in brain structure and psychopathology. Research in schizophrenia indicates that symptom-based approaches may be more promising, emphasizing a need for more transdiagnostic studies in clinical laterality research.

## Author Contributions

SO, JP, and AM conceptualized the manuscript, commented on the initial draft, and wrote the final draft of the manuscript. SO and AM wrote the initial draft of the manuscript. All authors contributed to the article and approved the submitted version.

## Conflict of Interest

The authors declare that the research was conducted in the absence of any commercial or financial relationships that could be construed as a potential conflict of interest.

## Publisher’s Note

All claims expressed in this article are solely those of the authors and do not necessarily represent those of their affiliated organizations, or those of the publisher, the editors and the reviewers. Any product that may be evaluated in this article, or claim that may be made by its manufacturer, is not guaranteed or endorsed by the publisher.

## Abbreviations

ADHD: attention deficit hyperactivity disorder
ALE: activation likelihood estimation
ASD: autism spectrum disorders
DTI: diffusion tensor imaging
FDR: false discovery rate
MDD: major depressive disorder
NODDI: neurite orientation dispersion and density imaging
OCD: obsessive–compulsive disorder
PTSD: posttraumatic stress disorder
RDoC: Research Domain Criteria
TBSS: tract-based spatial statistics.
